# Molecular mechanism of calcium permeability and magnesium block in NMDA receptors

**DOI:** 10.1038/s41593-026-02283-3

**Published:** 2026-05-05

**Authors:** Ruben Steigerwald, Max Epstein, Tsung-Han Chou, Noriko Simorowski, Hiro Furukawa

**Affiliations:** https://ror.org/02qz8b764 grid.225279.9 0000 0001 1088 1567 W.M. Keck Structural Biology Laboratory, Cold Spring Harbor Laboratory, Cold Spring Harbor, NY USA

**Keywords:** Cryoelectron microscopy, Ion channels in the nervous system

## Abstract

Hebbian neuroplasticity, which is thought to be a cellular substrate of learning and memory, can occur by means of coincidental detection of presynaptic neurotransmitter release and Ca^2+^ influx upon postsynaptic depolarization. This is mediated at a molecular level by *N*-methyl-D-aspartate-type glutamate receptors, which bind glutamate and glycine and facilitate Ca^2+^ influx upon relief of Mg^2+^ channel block during membrane depolarization. However, the structural mechanism underlying Ca^2+^ permeability and Mg^2+^ blockade in *N*-methyl-D-aspartate-type glutamate receptors has yet to be fully elucidated. Here we demonstrate using single-particle cryo-electron microscopy that Ca^2+^ permeation through the narrow constriction of the cation selectivity filter involves partial dehydration, as evidenced by several Ca^2+^ binding sites. In contrast, Mg^2+^ binds outside of the selectivity filter through a water network and remains hydrated, thereby acting as a channel blocker. Furthermore, the lipid network around the selectivity filter influences the stability of Mg^2+^ binding in a voltage-dependent manner. Our study details the transmembrane chemistry essential for initiating neuroplasticity.

## Abstract (web summary)

Using cryo-electron microscopy and electrophysiology, this study explains the structural basis for Ca^2+^ permeation and Mg^2+^ block in NMDA receptors and identifies a surrounding lipid network that may tune Mg^2+^-dependent voltage sensitivity.

## Main

The fundamental electrocellular event that initiates classic Hebbian plasticity for learning and memory is the coincidental detection of neurotransmitters and a voltage change in the postsynaptic membrane^1,2^. Postsynaptic *N*-methyl-D-aspartate-type glutamate receptors (NMDARs) are pivotally involved in this process. They facilitate high calcium influx upon glycine and glutamate binding^3^ when channel blockade by magnesium is relieved by depolarization^4,5^. This triggers downstream signaling only when both presynaptic and postsynaptic neurons are activated concurrently. Over the last four decades, extensive studies have elucidated the molecular and biophysical mechanisms underlying the voltage-dependent Mg^2+^ block of NMDARs^4–6^. A cluster of asparagine residues located deep within the channel pore, known as the QRN site^6^, plays a critical role in Mg^2+^ binding^6^. Both the association and dissociation of Mg^2+^ are strongly voltage dependent^7^, and the block is further modulated by divalent and monovalent cations^7–10^. Ca^2+^ permeability is governed initially by an extracellular motif, termed DRPEER^11^, but the asparagine residues in the QNR site are essential for the subsequent permeation step^6,12^. Despite the substantial insights gained from these studies, the structural mechanism by which the NMDAR channel pore distinguishes between calcium and magnesium ions remains unanswered. NMDARs are heterotetrameric receptors comprised of two glycine-binding GluN1 subunits and two glutamate-binding GluN2 subunits (GluN2A-D)^13^. NMDAR channel activation requires several layers of conditions, including occupancy of the GluN1 ligand-binding domain (LBD) with a co-agonist, glycine or D-serine, glutamate binding to the GluN2 LBD during neurotransmission and depolarization of the postsynaptic membrane to relieve the Mg^2+^ channel block at the transmembrane domain (TMD)^4,5^. The opening of NMDAR channels results in the conductance of Na^+^, K^+^ and Ca^2+^, triggering subsequent NMDAR-induced neuroplastic signaling through CaMKII activation^14,15^. The spatial-temporal regulation of Ca^2+^ influx in neurons controls various forms of plasticity^14,16^. Therefore, the molecular properties of Ca^2+^ permeation and voltage-dependent Mg^2+^ block in NMDAR are considered among the key drivers of Hebbian neuroplasticity in excitatory neurons. Despite advances in the structural biology of NMDARs over the last decade^17,18^, limited resolution in the transmembrane region has hindered understanding of how the pore recognizes Ca^2+^ as a permeant ion and Mg^2+^ as a blocker.

Here we address this long-standing question using single-particle cryo-electron microscopy (cryo-EM) with enhanced TMD resolution, revealing that Ca^2+^ occupies at least five binding sites within the cation selectivity filter—a process involving partial dehydration. In contrast, there are strictly two binding sites for hydrated Mg^2+^, at the extracellular and intracellular entrances of the selectivity filter. Finally, we demonstrate that the voltage sensitivity of the Mg^2+^ block can be modulated by residues surrounding a phospholipid (PL) network near the selectivity filter.

## Results

### Cryo-EM captures several calcium sites within the NMDAR channel pore

We first captured the calcium permeation pathway within the NMDAR by resolving structures of the GluN1a-2B NMDAR bound to agonists (glycine and glutamate), both in the presence of CaCl_2_ (10 mM) and in the absence of divalent cations (1 mM EDTA). We selected the GluN1a-2B NMDAR subtype, as it exhibits the highest Ca^2+^ permeability and sensitivity to Mg^2+^ block, similar to the GluN1a-2A NMDAR.

**Fig. 1:**
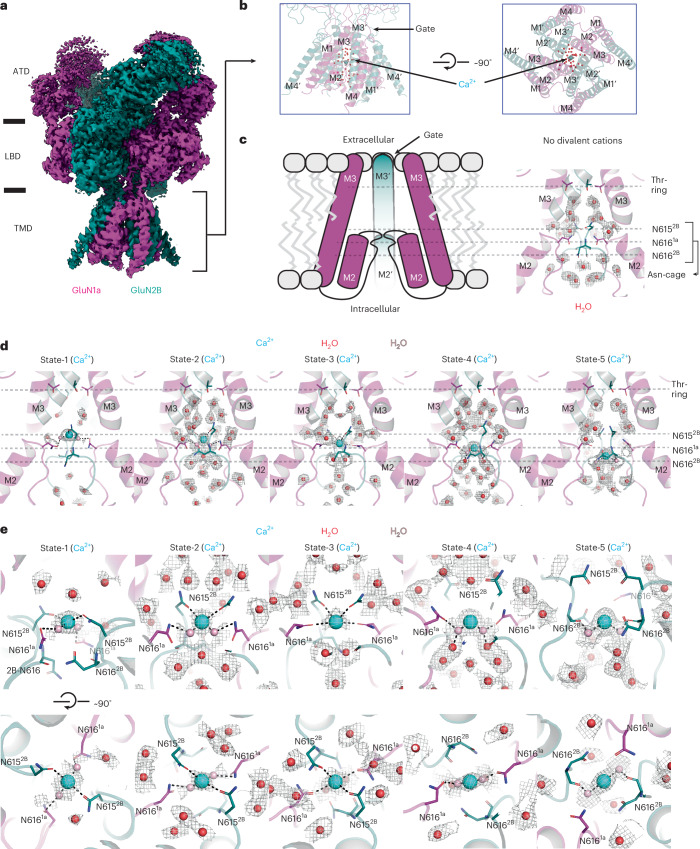
Structure of GluN1a-2B NMDAR in complex with agonists and calcium ions. **a**,**b**, Cryo-EM density (**a**) and the TMD model (**b**) of the GluN1a-2B NMDAR bound to agonists and Ca^2+^. One of the five Ca^2+^ (cyan sphere) positions and water molecules (red spheres) in the pore is shown. ATD, amino-terminal domain. **c**, Schematic figure of the pore region and a zoom-in view of the agonists-bound GluN1a-2B NMDAR with no divalent cation (EDTA-condition) showing cryo-EM density (gray mesh) for water molecules. The Thr-ring is a part of the channel gate, whereas the Asn-cage constitutes the cationic selectivity filter. **d**, Views of the channel pore with Ca^2+^ at five distinct positions (State-1 to State-5) from the top to the bottom of the Asn-cage. **e**, Close-up views of the Ca^2+^ sites viewed from the side and top of the Asn-cage. Pink spheres: putative water molecules coordinating Ca^2+^ directly, modeled based on continuous density within 2.5 Å of Ca^2+^. Dotted lines: polar interactions.

**Extended Data Fig. 1:**
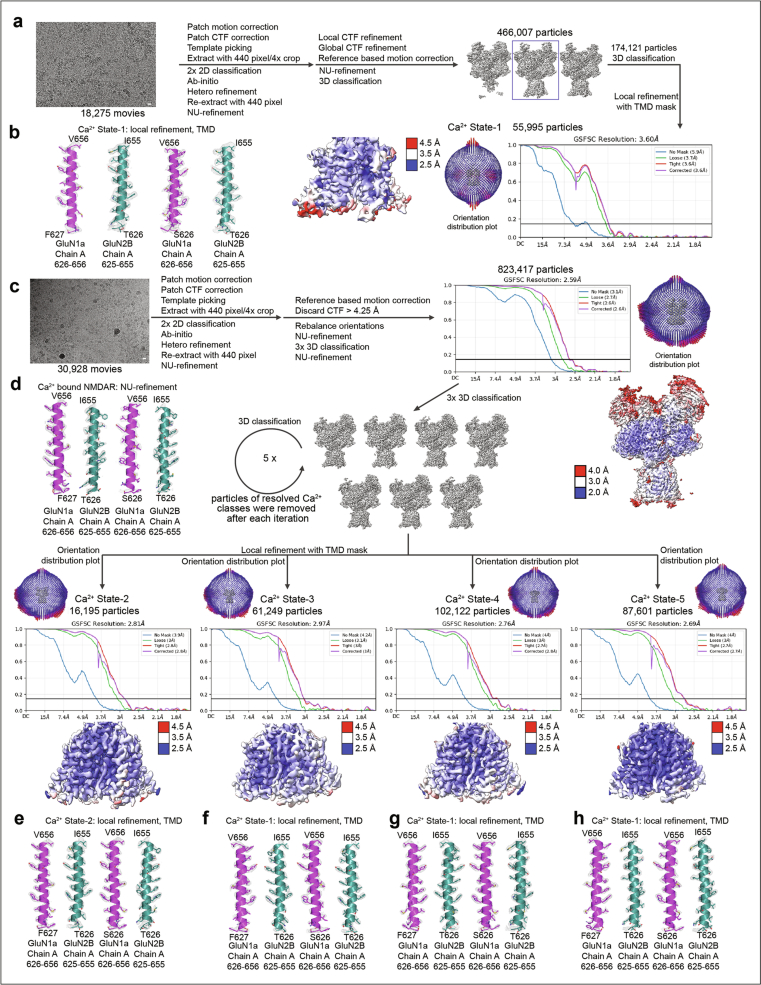
Single-particle cryo-EM of the Ca^2+^-bound GluN1a-2B NMDAR (Related to Fig. 1). **a**, Single-particle cryo-EM workflow of the Ca^2+^ State-1 GluN1a-2B NMDAR. **b**, Map quality assessment of the Ca^2+^ State-1 GluN1a-2B NMDAR at the M3/M3’ region. **c**, Single-particle cryo-EM workflow of the Ca^2+^ State-2-5 GluN1a-2B NMDAR. **d**, Map quality assessment of Ca^2+^ bound GluN1a-2B NMDAR NU-refinement at the M3/M3’ region. **e-h**, Map quality assessment of the Ca^2+^ State-2-5 GluN1a-2B NMDAR at the M3/M3’ region.

**Extended Data Fig. 2:**
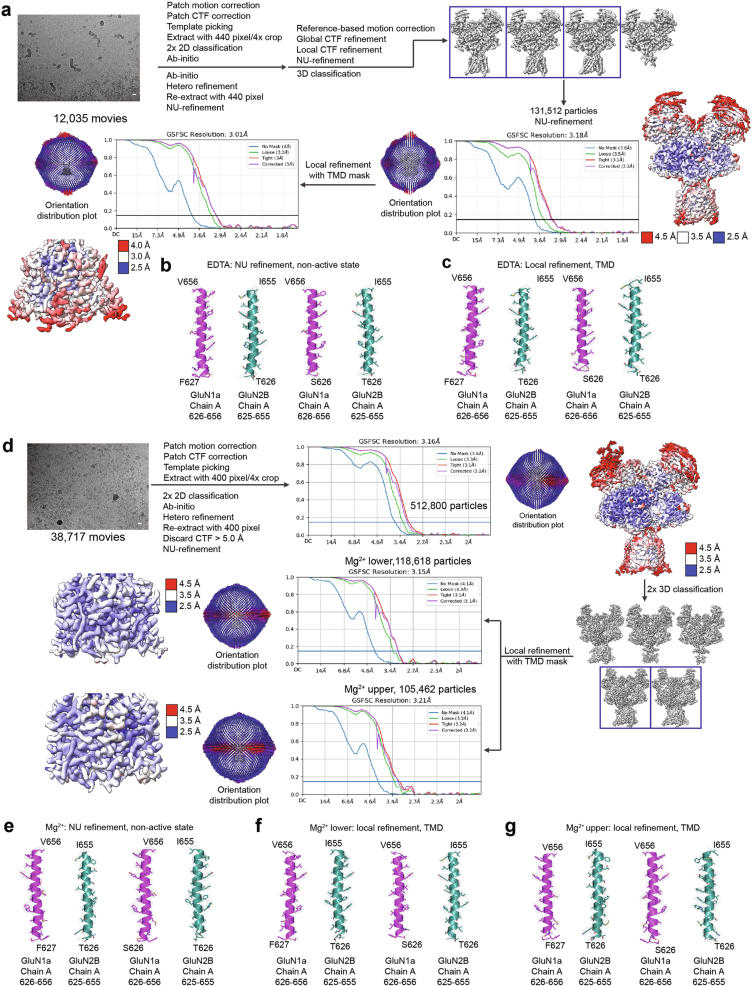
Single particle cryo-EM of divalent cation-free EDTA-treated, and the Mg^2+^-bound GluN1a-2B NMDAR (Related to Fig. 1 and Fig. 2). **a**, Single-particle cryo-EM workflow of the divalent cation-free EDTA-treated GluN1a-2B NMDAR. **b**, Map quality assessment of the divalent cation-free EDTA-treated GluN1a-2B NMDAR NU-refinement at the M3/M3’ region. **c**, Map quality assessment of the divalent cation-free EDTA-treated GluN1a-2B NMDAR TMD local refinement at the M3/M3’ region. **d**, Single-particle cryo-EM workflow of the Mg^2+^-bound GluN1a-2B NMDAR. **e**, Map quality assessment of the Mg^2+^-bound GluN1a-2B NMDAR NU-refinement at the M3/M3’ region. **f**-**g**, Map quality assessment of the Mg^2+^-bound GluN1a-2B NMDAR TMD local refinement at the M3/M3’ region.

Our single-particle cryo-EM structures achieved TMD resolution ranging from 2.3 Å to 3.0 Å by combining extensive three-dimensional (3D) classification followed by local refinement of the TMD channel region (Fig. 1a,b, Extended Data Fig. 1 and Supplementary Table 1). This allowed us to monitor Ca^2+^ and tightly bound water oxygens at distinct positions in the pore (Fig. 1 and Extended Data Fig. 1). The single-particle analysis strategy implemented here (Extended Data Fig. 1) was crucial in unveiling the hydration pattern of the NMDAR channel pore and Ca^2+^ at several positions (Fig. 1d,e). Our analysis revealed Ca^2+^ ions within the narrow constriction formed by the re-entrant loops located between the M2 and M3 helices of GluN1a and the M2′ and M3′ helices of GluN2B (Fig. 1b). This motif contains a cluster of Asn (N) residues, classically referred to as the QRN site^6^, which plays a key role in determining the calcium permeability pattern in ionotropic glutamate receptors (iGluRs) (Fig. 1c)^6,19–21^. For example, the equivalent sites are Gln (Q) or Arg (R) in AMPARs and kainate receptors. In AMPARs, the GluA2 subunit has an arginine at this position—a feature that disfavors Ca^2+^ permeability. However, channels spanning a range of Ca^2+^-permeability have been observed when GluA2 co-assembles with GluA1 (harboring glutamine) together with auxiliary subunits, including CNIH and TARP γ-2 (ref. ^22^). The QRN site in the GluN1a-2B NMDAR has a narrow cage-like structure formed by the side chains of GluN1a Asn616 and main and side chains of GluN2B Asn615-616 (Asn-cage; Fig. 1c). No equivalent density was observed in samples where calcium was not included and divalent cations were chelated by EDTA (Fig. 1c (‘No divalent cations’) and Extended Data Fig. 2a–c), indicating that the observed cryo-EM densities represent Ca^2+^ ions.

The Ca^2+^ ions occupy five positions within the Asn-cage, revealing the permeation pathway (Fig. 1d,e and Supplementary Video 1). Here we arbitrarily name them State-1 to State-5, from the extracellular to the intracellular side. In States-1, -2 and -3, Ca^2+^ ions are located around the upper entry site of the Asn-cage, constituted by the two GluN2B Asn615 residues, which form the narrowest constriction within the NMDAR channel. The distances between Ca^2+^ and GluN2B Asn615 residues are sufficiently short to allow direct interactions with a partially dehydrated Ca^2+^ ion (Fig. 1d,e; State-1 to State-3, dotted lines). We speculate that, based on continuous density with Ca^2+^ and interacting residues, the hydration shell waters (pink spheres) make secondary contacts with GluN1a Asn616 as well (Fig. 1d,e; State-1 and State-2), whereas in State-3, Ca^2+^ is positioned to form direct interactions with both GluN2B Asn615 and GluN1a Asn616 (Fig. 1d,e; State-3). In State-4 and State-5, the interactions are through secondary interactions of the Ca^2+^ ion hydration shells with GluN1a Asn616 side chain (State-4) or the GluN2B Asn616 main chain carbonyl (State-5) (Fig. 1d, e; State-4 and State-5). Above and beneath the Asn-cage motif, there is sufficient space for hydrated Ca^2+^ ions to diffuse; therefore, no defined Ca^2+^ density was observed. Based on the structural observations of Ca^2+^ in various positions, we speculate that the partial dehydration of the Ca^2+^ ion, mainly by GluN2B Asn615, is required for Ca^2+^ to pass through the Asn-cage (Fig. 1 and Supplementary Video 1).

Although we focused on analyses of the TMD region, the predominant overall protein conformation here is the nonactive state, where glycine and glutamate are bound to the GluN1 and GluN2B LBDs, respectively. Meanwhile, the gate at the entrance of the TMD channel, formed by the SYTANLAAF^23^ and VIVI^24–26^ motifs, is closed^27–30^. Our recent structural studies have shown that the region surrounding the Asn-cage, located below (toward the cytoplasmic domain), remains unchanged or minimally altered between open and closed states^24,25^. Therefore, although our structure shows a closed gate at the extracellular entrance, the architecture of the cation-permeation pathway remains intact, thereby supporting the calcium permeation mechanism through the Asn-cage.

### Two magnesium binding sites around the selectivity filter

**Fig. 2:**
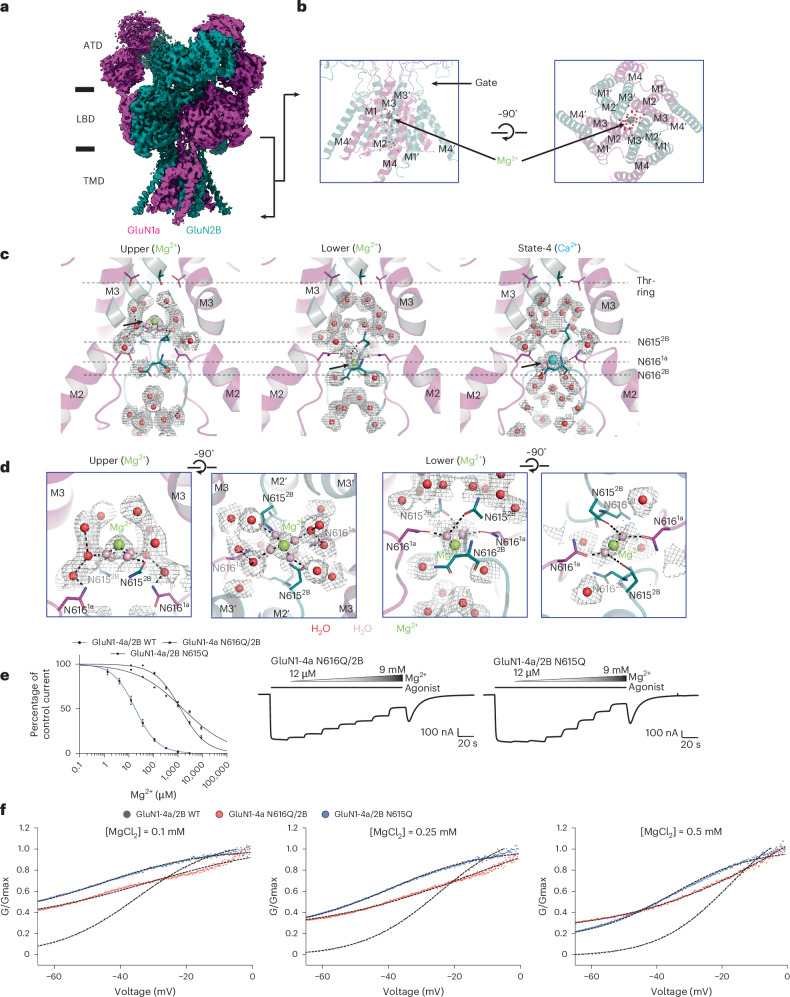
Structure of GluN1a-2B NMDAR in complex with agonists and magnesium ions. **a**,**b**, Cryo-EM density (**a**) and models showing the upper Mg^2+^ (lime sphere) and water molecules (red spheres) in the pore (**b**). **c**, View of the channel pore in complex with two Mg^2+^ sites, Upper and Lower, in comparison with Ca^2+^ at the State-4 position (arrows). **d**, Zoomed-in view of the Upper and Lower Mg^2+^ binding sites viewed from the side and top of the Asn-cage. Pink spheres: plausible directly coordinating water molecules with Mg^2+^, modeled based on continuous density with Mg^2+^. Dotted lines: polar interactions. **e**, Concentration-responses of Mg^2+^ on wild type (WT) and Asn-cage mutants at −60 mV holding potential. Left panel: data points (mean values ± s.d. from independent oocytes). Right panels: representative recording traces (leftmost trace: WT, *n* = 5, GluN1a (Asn616Gln); rightmost trace: *n* = 4, GluN2B (Asn615Gln)). **f**, G/V plot analysis of Mg^2+^ block at three Mg^2+^ concentrations. Data plots are fitted with the modified Boltzmann equation (dashed lines; Methods). All currents were induced by the application of glycine and glutamate at 100 µM.

To understand the mechanism of channel blockade by Mg^2+^, we obtained a cryo-EM structure of the GluN1a-2B NMDAR in the presence of agonists (glycine and glutamate) and MgCl_2_ (Fig. 2a,b, Extended Data Fig. 2 and Supplementary Table 1). As for the study of the Ca^2+^-bound structures described above, the cryo-EM sample was prepared in the presence of agonists to promote the opening of the VIVI-gate and SYTANLAAF-gate^24,25^, allowing Mg^2+^ entry from the extracellular side (Fig. 2b). Single-particle cryo-EM captured predominantly the nonactive, closed gate conformation, within which the Mg^2+^ ions are trapped in the pore. This may have been possible since we retained 150 mM NaCl in the sample, which has been shown to slow down the unblocking of Mg^2+^ (refs. ^31,32^). Here we implemented a similar single-particle analysis strategy to that used in the study of Ca^2+^-bound structures, including extensive 3D classification followed by local refinement of the TMD to achieve a resolution ranging from 2.7 Å to 3.0 Å, thereby allowing us to monitor the binding of Mg^2+^ and associated water molecules (Extended Data Fig. 2d–g). Unlike the Ca^2+^-bound structures, where five binding sites were observed, there are strictly just two distinct Mg^2+^ binding sites, one above the Asn-cage (upper) and the other at the intracellular side of the Asn-cage (lower), which we speculate as extracellular and intracellular Mg^2+^ blocking sites, respectively (Fig. 2c,d). These densities are absent in the sample without added divalent metals (Fig. 1c) and are distinct from the Ca^2+^-bound structures. The upper site Mg^2+^ does not contact the NMDAR protein directly as the closest residues, GluN2B Asn615s (side chain oxygens), are 4.1 Å away. Instead, the hydrated Mg^2+^ is in contact with a surrounding water molecule network, interacting with GluN2B Asn615 (Fig. 2c). The Mg^2+^ hydration shell further interacts with surrounding water molecules, which form a hydrogen bond network with the GluN1a Asn616 residues (Fig. 2c,d). Therefore, the binding of the extracellular Mg^2+^ involves a water-mediated network with GluN1a Asn616 and GluN2B Asn615 (Fig. 2c,d). It is important to note that, unlike Ca^2+^, Mg^2+^ does not seem to traverse the Asn-cage, as no positions analogous to the several Ca^2+^ sites are observed. It has been well documented that the dehydration of Mg^2+^ requires substantially more energy than that of Ca^2+^ due to the smaller ionic radius of Mg^2+^, which leads to stronger attraction to the oxygen atoms of water and higher hydration energy^33^. Since passing through the narrow Asn-cage would require dehydration, we suggest that a higher dehydration energy requirement for Mg^2+^ may be the factor that disfavors the permeation and makes Mg^2+^ a blocker right above the two GluN2B Asn615 residues. Consistent with our structural observation as well as previous reports^6,34^, the GluN1a Asn616Gln and GluN2B Asn615Gln mutations robustly affect the extracellular Mg^2+^ block, altering half-maximal inhibitory concentration (IC_50_) values (Fig. 2e), voltage dependency (represented by *δ* in the modified Boltzmann fit), and half-block voltage (V_50_) (Fig. 2f, Supplementary Table 2 and Methods).

The Mg^2+^ ion bound at the lower site interacts with GluN1 Asn616 and GluN2B Asn616 through a network of water molecules (Fig. 2c,d (Lower)). This Mg^2+^ binding position is similar to State-4 in the Ca^2+^ structure (Fig. 2c (State-4)), although the binding modes differ from each other (Figs. 1c,d (State-4) and 2c,d (Lower)). Given that hydrated Mg^2+^ is too large to pass through the Asn-cage from its extracellular entrance, we speculate that the lower site represents the intracellular Mg^2+^ block site, which is accessible from the intracellular side^8,35^. As our structure shows, the region outside the Asn-cage extending toward the intracellular exit is wide open; therefore, Mg^2+^ has free access to the intracellular entrance of the Asn-cage. Our observation of distinct extracellular and intracellular Mg^2+^ sites is consistent with previous suggestions based on the additive effects of the two in electrophysiological experiments^8,35^. Although channel blockade by extracellular Mg^2+^ is relevant to neuroplasticity, the role of blockade by intracellular Mg^2+^ remains unclear.

### A lipid network surrounds the NMDAR pore and affects voltage-dependent Mg^2+^ block

**Fig. 3:**
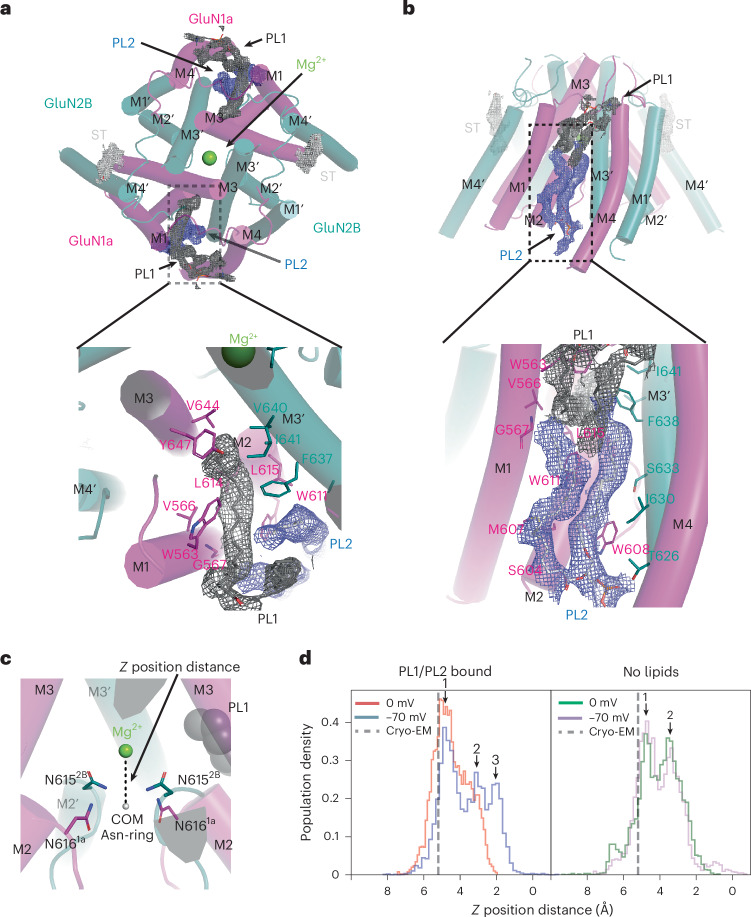
Lipid binding sites in GluN1a-2B NMDAR. **a**,**b**, Cryo-EM captures the tightly bound lipids in the TMD viewed from the extracellular side (**a**) and side of the membrane (**b**). Cryo-EM density for sterol-like density (ST, white mesh) around GluN2B M4′ and PL1 (dark gray mesh) and PL2 (slate mesh). Note that one of the acyl chains of PL1 wedges into the back of the upper Mg^2+^ binding site (lime). **c**,**d**, MD simulations estimating the population density of *Z* position distance distribution between Mg^2+^ and the center of mass of GluN1 Asn616 and GluN2B Asn615 Cα atoms (**c**) at 0 mV and −70 mV in the presence (**d**, left) and absence (**d**, right) of PL1 and PL2. One of the stable positions identified by MD simulations, peak 1, corresponded closely to the cryo-EM-derived position (dashed line). At −70 mV, additional peaks (peaks 2 and 3) emerged at shorter *Z* position distances relative to 0 mV, suggesting voltage-dependent alterations in Mg^2+^ dynamics in the presence of PL1 and PL2 (**d**, left). By contrast, no such voltage-dependent shift was observed in the absence of these lipids, with population density distributions appearing similar between 0 mV and −70 mV (**d**, right). Histograms were constructed using 50 bins across the *Z* position distance range.

Our workflow in single-particle cryo-EM allowed us to capture two lipid-binding sites in the NMDAR complex. In all three conditions, ‘no added divalent cations’, ‘Ca^2+^-bound’ or ‘Mg^2+^-bound’, two PLs (PL1 and PL2) that are networked with one another are observed at each GluN1a–GluN2B interface in the tetramer symmetrically (Fig. 3a,b). Although the phosphate headgroup of PL2 is visible, that of PL1 exhibits a weak density. Thus, it remains possible that the PL1 density reflects the lauryl maltose neopentyl glycol (LMNG) detergent used during purification. Nonetheless, a prominent hydrophobic pocket capable of accommodating an alkyl chain is evident at the PL1 site. PL1 and PL2 are located near the ‘back’ of the Asn-cage where Mg^2+^ block occurs (Fig. 3a,b). One acyl chain from PL1 is placed almost parallel to the membrane plane and is surrounded by GluN1a M1, M2 and M3 and GluN2B M3′, and the other acyl chain is placed next to GluN1a M4 (Fig. 3a). The two acyl chains of PL2 are placed orthogonal to the membrane plane and are surrounded by GluN1a M1 and M2 and GluN2B M3′ (Fig. 3b). One of the two PL1 acyl chains wedges into a pocket and interacts with GluN1a Leu615, Trp611, Met607, Ser604 and GluN2B Phe637 and Ile641 (Fig. 3a). Residues such as GluN1a Met607, Ser604, Trp611 and GluN2B Ile630 and Ser633 interact with PL2 (Fig. 3b). PL1 and PL2 are associated tightly with each other and integrated into the protein structure as a single entity (Fig. 3a,b). Due to their proximity to the ‘back’ of the Asn-cage, we hypothesized that these lipid-binding sites may affect Mg^2+^ binding through subtle effects on mobility of the coordinating asparagine residues.

To explore this possibility, we analyzed the binding stability of the upper Mg^2+^ ion responsible for the voltage-dependent channel block by extracellular Mg^2+^ by calculating the distance between the Mg^2+^ ion and the center of geometry of the c-α atoms of GluN1a Asn616 and GluN2B Asn615 in the presence and absence of lipids (PL1 and PL2) and at resting (−70 mV) and depolarized (0 mV) membrane voltages by molecular dynamics (MD) simulations (Fig. 3c,d). Although the branching acyl chains of the phospholipids are visible, there was insufficient cryo-EM density to identify the headgroup definitively. Here phosphatidylcholine was modeled arbitrarily as a lipid. Our simulations showed that Mg^2+^ can move on the *Z* axis but not pass the entrance of the Asn-cage, consistent with the notion that Mg^2+^ blocks the channel (Fig. 3d). In the presence of lipids, Mg^2+^ can move closer to the Asn-cage more readily at −70 mV (blue) than 0 mV (orange), indicative of preferential binding of Mg^2+^ at the resting potential of neurons (Fig. 3d; left panel, arrows). In the absence of lipids, the distribution of Mg^2+^ positions at 0 and −70 mV is similar, suggesting decreased voltage sensitivity (Fig. 3d; right). Thus, our MD simulations support the involvement of bound lipids in the voltage-sensitive Mg^2+^ block.

### Mutations on lipid-binding residues affect Mg^2+^ block

**Fig. 4:**
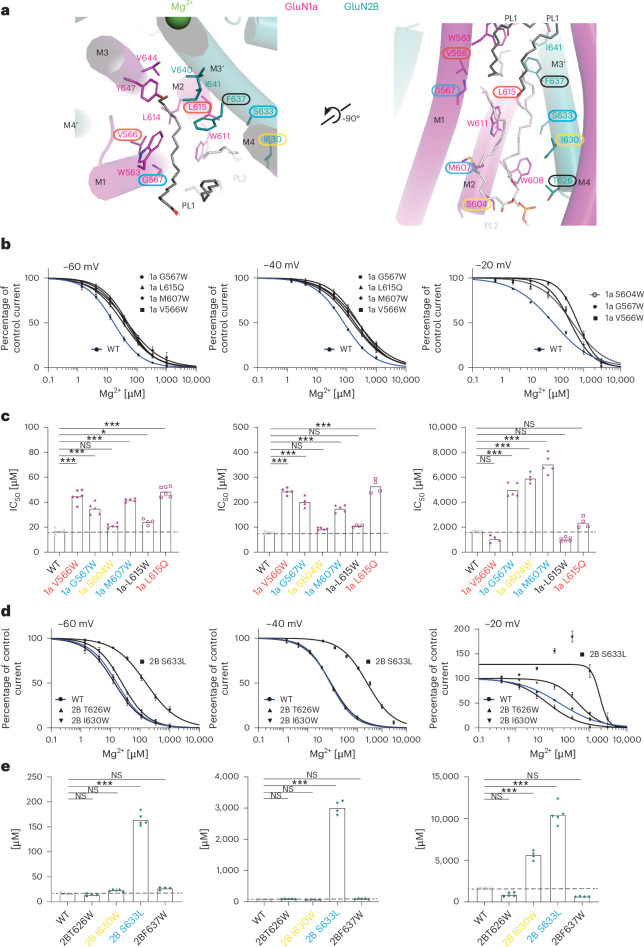
Effects of lipids on voltage-dependent Mg^2+^ block in GluN1a-2B NMDAR. **a**, Close-up views of PL1 (dark gray sticks) and PL2 (white sticks) binding sites from the top (left) and side (right) of the membrane. Residues enclosed by cyan, orange and yellow ovals have altered Mg^2+^ sensitivity when mutated at all tested voltages, at −60 mV and −40 mV, and only at −20 mV, respectively. The residues outlined in black show no mutational effect at any voltage. **b**,**d**, Mg^2+^ concentration-response curves for GluN1a (**b**) and GluN2B (**d**) mutants. Data points represent the mean values ± s.d. from independent oocytes (WT: −60 mV *n* = 5, −40 mV *n* = 5, −20 mV *n* = 6; GluN1a L615Q: −60 mV *n* = 6, −40 mV *n* = 4, −20 mV *n* = 4; M607W −60 mV, −40 mV and −20 mV: *n* = 5; V566W: −60 mV *n* = 6, −40 mV *n* = 5, −20 mV *n* = 4; G567W: −60 and −20 mV *n* = 5, −40 mV *n* = 4; S604W: −60 and −40 mV *n* = 5, −20 mV *n* = 4, GluN2B T626W −60 mV *n* = 4, −40 and −20 mV *n* = 5; I630W: −60 mV *n* = 5, −40 and −20 mV *n* = 4; S633L −60 and −20 mV *n* = 4, −40 mV *n* = 5). **c**,**e**, Corresponding mean IC_50_ values ± s.d. derived from the TEVC recording at −60m V, −40 mV and −20 mV for the GluN1a (**c**) and GluN2B (**e**) mutants. Color codes as in **a**. The GluN1 subunit used in these TEVC experiments is GluN1-4a. Statistical significance in **c** and **e** was assessed using one-way analysis of variance followed by Dunnett’s test (two-sided) comparing each group with the WT. *P* values were adjusted for multiple comparisons with family-wise α = 0.05. ***P < 0.001, **P = 0.001 to < 0.01, *P = 0.01 to < 0.05. If no asterisk noted, the result is not significant (NS); exact *P* values are provided in Supplementary Table 3.

**Extended Data Fig. 3:**
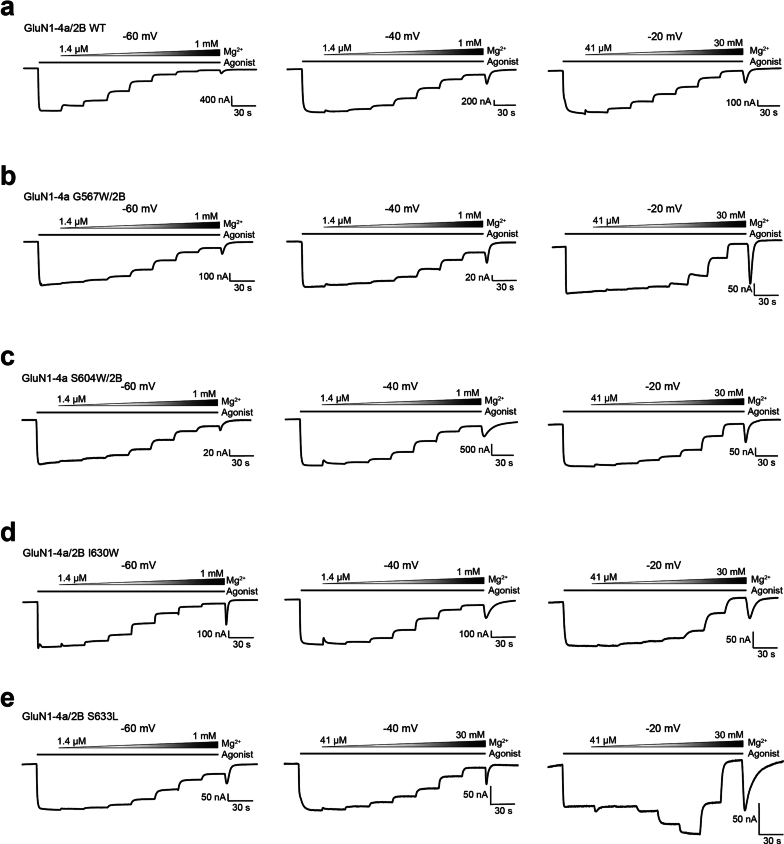
IC_50_ evaluation of the Mg^2+^-block on GluN1a-2B NMDAR lipid pocket mutants (related to Fig. 4). **a**-**e**, IC_50_ evaluation through TEVC electrophysiology on cRNA-injected (**a**: GluN1a-2B NMDAR wild-type, **b**: GluN1a Gly567Trp/2B NMDAR, **c**: GluN1a Ser604Trp/2B NMDAR, **d**: GluN1a/2B Ile630Trp NMDAR, **e**: GluN1a/2B Ser633Leu NMDAR) oocytes at −60, −40, and −20 mV. The isotype of GluN1a used in these TEVC experiments is GluN1-4a.

To further assess the involvement of PL1 and PL2 in Mg^2+^ block, we incorporated site-directed mutations into the lipid-binding pockets (Fig. 4a; mutated residues are highlighted by ovals) and evaluated their effects on the potency of Mg^2+^ block using two-electrode voltage clamp (TEVC) electrophysiology (Fig. 4b–e). These point mutants were designed to perturb the binding of PL1, PL2 or both. Concentration-response curves for Mg^2+^ block at three different voltages, −60 mV, −40 mV and −20 mV, from which IC_50_ values were calculated and compared with those of wild type GluN1a-2B NMDAR (Fig. 4b–e, Extended Data Fig. 3 and Supplementary Table 3). The effects of all GluN1a mutants were to increase the Mg^2+^ IC_50_ values (decreased potency) and could be grouped into three categories, significant changes at all voltages (Gly567Trp, Met607Trp; cyan in Fig. 4a,c), significant effect at −60 mV and −40 mV but not at −20 mV (Val566Trp and Leu615Gln, dark orange in Fig. 4a,c), and no effect at −60 mV and −40 mV but a significant effect at −20 mV (Ser604Trp, gold in Fig. 4a,c). Furthermore, Leu615Trp did not exhibit any significant effect at any voltage (Fig. 4b,c). For GluN2B, Ser633Leu at all voltages lowered Mg^2+^ potency considerably (cyan in Fig. 4a,d), Ile630Trp showed a significant effect at −20 mV, but not at −60 mV and −40 mV (gold in Fig. 4a,d), whereas Thr626Trp and Phe637Trp had no significant impact (Fig. 4d,e). Mutations that target PL2 or both PL1 and PL2 tend to affect Mg^2+^ block at all voltages (1a-Gly567Trp, 1a-Met607Trp and 2B Ser633Leu, cyan in Fig. 4a,c,d). The mutations near the headgroup of PL2 (1a-Ser604Trp and 2B-Ile630Trp, gold in Fig. 4a,c,d) tend to affect Mg^2+^ potency only at −20 mV. The mutations that target only PL1 (dark orange in Fig. 4a,c,d) tend to affect Mg^2+^ potency at −60 and −40 mV but not at −20 mV. The GluN2B Ser633Leu mutation was previously reported to alter the potency of Mg^2+^ block as well as Ca^2+^ permeability^36,37^. It is important to note that GluN2B Ser633 is located at the PL2 binding site, and we anticipate that the GluN2B Ser633Leu mutant interacts with PL2 differently. At the voltages tested in the experiments above, we did not observe a pronounced effect of Mg^2+^ potentiation on the GluN1a-2B NMDAR^38,39^ in most mutants. GluN2B Ser633Leu showed robust Mg^2+^ potentiation at −20 mV (Fig. 4d).

**Extended Data Fig. 4:**
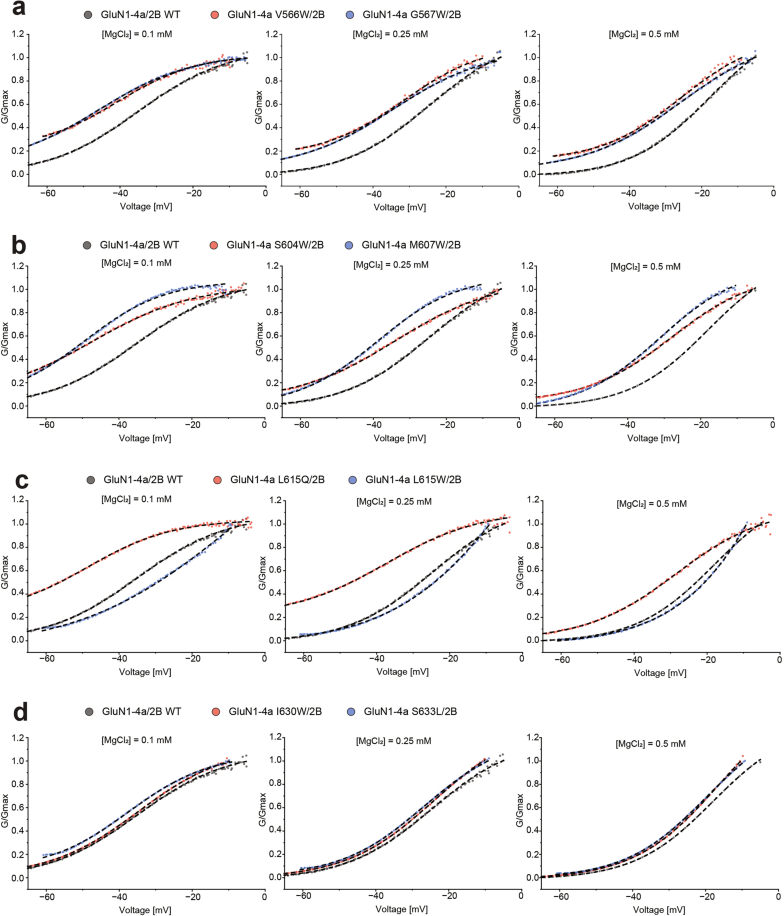
G/V plots of Mg^2+^-block on GluN1a-2B NMDAR lipid pocket mutants (related to Fig. 4). G/V plot analysis of Mg^2+^ block at three Mg^2+^ concentrations (0.1, 0.25, and 0.5 mM). The four groups (**a**-**d**) of two mutants (orange and blue) are tested and compared with the wildtype (gray). The normalized values (G/Gmax) are plotted against voltage (mV). The G/V plots are fitted with the modified Boltzmann equation (dashed lines, Methods).

**Extended Data Fig. 5:**
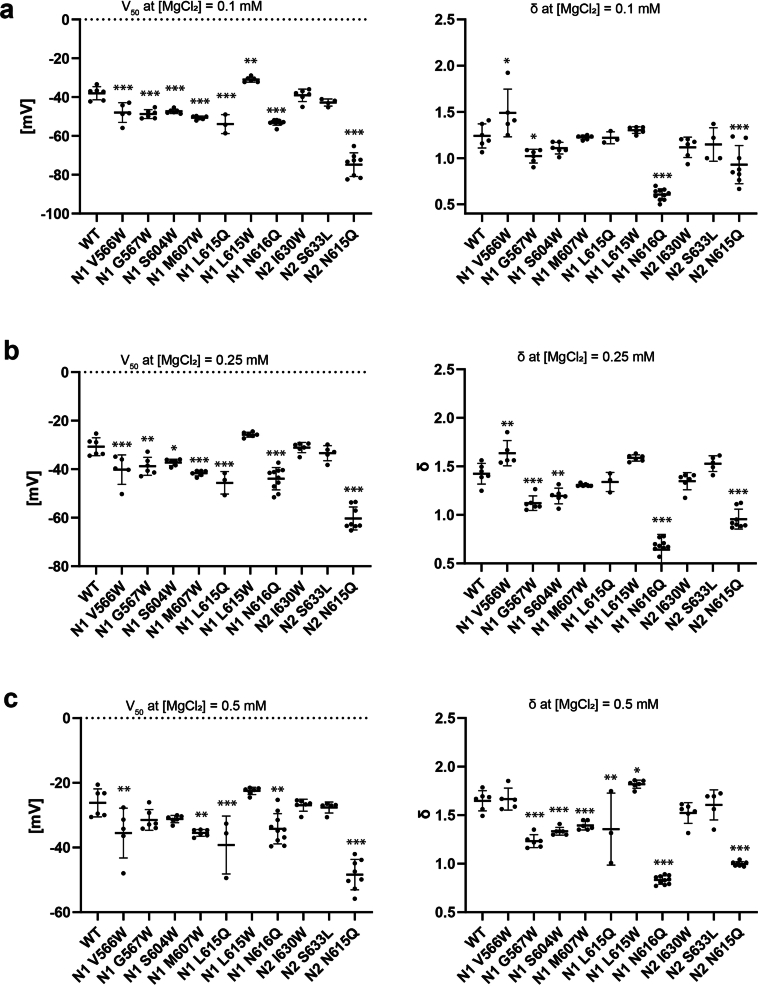
Comparison of V50 and δ values between the lipid-pocket mutants and wildtype. (related to Fig. 4). **a**-**c**, mean V_50_ and δ values ± SE of the lipid pocket mutants, calculated from G/V plot analyses, are plotted and compared with those of wildtype (WT) at three different MgCl_2_ concentrations (**a**: 0.1 mM, **b**: 0.25 mM, and **c**: 0.5 mM). Statistical significance was assessed using one-way analysis of variance (ANOVA) followed by Dunnett’s test (two-sided) comparing each group with the WT. P values were adjusted for multiple comparisons with family-wise α = 0.05. (*** p < 0.001, ** 0.001<p < 0.01, * 0.01<p < 0.05, if no asterisk is noted the result is not significant; exact p values are provided in Extended Data Table 4). Data points represent the mean values ± SE from independent oocytes; WT n = 6; GluN1a: V566W n = 5, G567W n = 7, S604W n = 6, M607W n = 6, L615W n = 6, L615Q n = 3, N616Q n = 10; GluN2B: I630W n = 7, S633L n = 4, N615Q n = 8. GluN1-4a and GluN2B are labelled as N1 and N2, respectively, on the X-axis.

To further characterize the mutational effects on voltage-dependent Mg^2+^ block, we performed G/V analysis of these mutants at three Mg^2+^ concentrations (0.1 mM, 0.25 mM and 0.5 mM) (Extended Data Figs. 4–5 and Supplementary Table 4). We observed significant shifts in the V_50_ values toward more negative voltages for GluN1a Val566Trp, Met607Trp, and Leu615Gln, but no change in the δ values. Notably, GluN1a Gly567Trp and Ser604Trp showed V_50_ values that were more negative and smaller δ values (except at 0.1 mM Mg^2+^) than the wild type. No significant differences in V_50_ and δ were detected in the GluN1a Leu615Trp and the GluN2B mutants. Although GluN1a Gly567Trp and Ser604Trp mutants exhibit lower voltage dependency, indicated by smaller δ values, this change was less pronounced than in GluN2B Asn615Gln, which participates directly in Mg^2+^ binding (Fig. 2).

**Extended Data Fig. 6:**
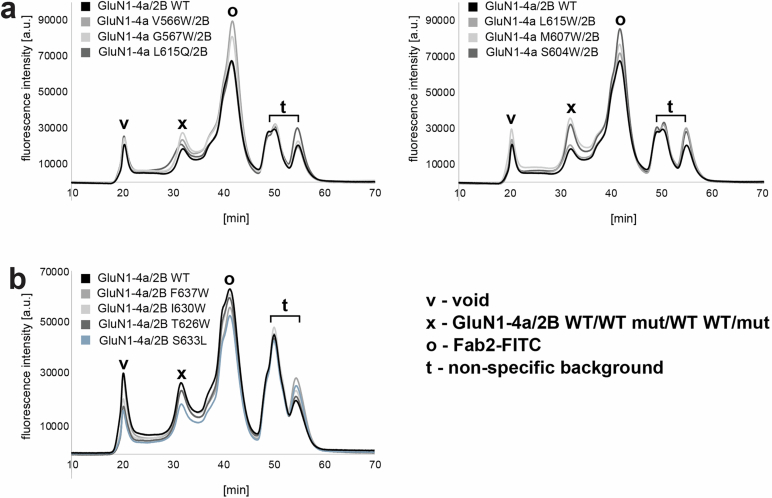
FSEC analyses on the GluN1a-2B NMDAR lipid pocket mutant proteins (related to Fig. 4). FSEC analyses of the lipid-pocket mutant NMDAR proteins. The NMDAR proteins transiently expressed in HEK293 cells are detected by anti-GluN2B Fab2 labelled with FITC. Superose6 was used for SEC, and 494 nm (excitation)/521 nm (emission) was used to measure fluorescence intensity (a.u. – arbitrary unit). **a**, Comparison of wildtype (WT, black trace) and GluN1-4a mutants (grays). **b**, Comparison of WT and GluN2B mutants (grays and light blue). Peak x in all traces represents the intact GluN1-4a/2B tetramer proteins.

Finally, we tested the effects of the mutations on stability and oligomeric assembly pattern using a modified fluorescence-coupled size-exclusion chromatography (SEC) (FSEC)^40,41^. To achieve this, we first prepared an anti-GluN2B antibody, Fab2 (ref. ^42^), labeled with fluorescein isothiocyanate (FITC). We expressed wild type and mutant GluN1a-2B NMDARs in HEK293 cells by transient expression and solubilized the protein by LMNG. The solubilized lysate and the FITC-labeled Fab2 were analyzed for oligomeric assembly and sample homogeneity by assessing retention times and peak shapes as references. Our FSEC experiments show sharp peaks at 32 min (Extended Data Fig. 6), representing tetramers for both the wild type and mutant proteins. The mutations did not cause loss of the tetrameric peak or peak broadening, indicating that the functional alteration caused by the mutations is not due to protein misfolding, but probably a local structural change.

## Discussion

**Fig. 5:**
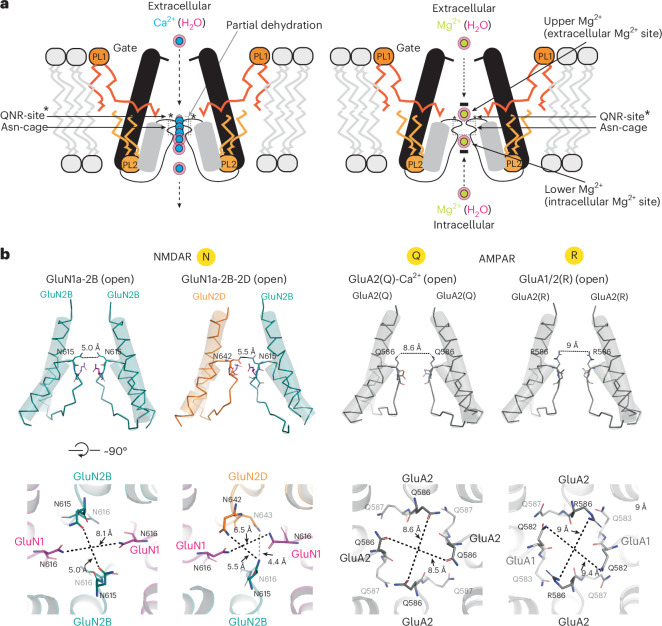
Molecular mechanism of Ca^2+^ permeability and Mg^2+^ block in NMDAR and comparison with AMPARs. **a**, Cation permeation through the narrow Asn-cage (dotted rectangles) requires partial dehydration. Hydrated Ca^2+^ (concentric cyan and light pink) can undergo partial dehydration as it permeates through the Asn-cage. In contrast, Mg^2+^ requires substantially higher energy for dehydration, making its permeation through Asn-cage energetically unfavorable. Instead, hydrated Mg^2+^ (concentric lime and light pink) binds at upper and lower sites through a structured network of water molecules, corresponding to the extracellular and intracellular Mg^2+^ block sites, respectively. Note that extracellular Mg^2+^ can access the upper site only when the channel gate is open in response to agonists. The voltage sensitivity of the extracellular Mg^2+^ block may be regulated partly by residues surrounding the tightly bound lipids, PL1 and PL2 (dark and light orange). **b**, Side views of open channel structures for GluN1a-2B (PDB code: 9ARE, N), GluN1a-2B-2D (PDB code: 9D38, Q), GluA2(Q)/TARP gamma-2 (PDB code: 8FP9, R) and GluA1/2 (R)/TARP gamma-8 (PDB code: 7OCF). TARP gamma-2 and gamma-8 are omitted for visual comparison with NMDARs. Note that the pore dimensions of the QRN sites are substantially wider in AMPARs than in NMDARs, suggesting the possibility that Ca^2+^ permeation may occur without dehydration in AMPARs.

Ca^2+^ permeation and voltage-dependent channel blockade by extracellular Mg^2+^ are two fundamental molecular properties of NMDARs that are crucial to neural plasticity. Our cryo-EM structures captured Ca^2+^ at five positions throughout the cation selectivity filter (Asn-cage) and Mg^2+^ at two distinct positions above and in the lower part of the Asn-cage. Based on our cryo-EM structures, we propose that Ca^2+^ permeability is facilitated by the ability of the NMDAR Asn-cage to directly coordinate with and partially dehydrate Ca^2+^ (Fig. 5a). In contrast, the NMDAR Asn-cage is unable to dehydrate Mg^2+^—a process that would incur a substantially higher energetic cost compared to Ca^2+^. Consequently, Mg^2+^ cannot traverse the Asn-cage and instead forms a water-mediated network that enables it to act as a channel blocker at physiological membrane potentials (Fig. 5a). It is worth noting that this was presciently proposed by Ascher and Nowak, and by Mayer and Westbrook, well before the structures of any channel were available^5,7,9^. In the current study, we identified two Mg^2+^ binding sites, upper and lower, which probably correspond to the extracellular and intracellular Mg^2+^ blockade sites, respectively. The presence of agonists in our cryo-EM samples, which promotes channel opening^24,25^, allows Mg^2+^ ions to access the ‘Upper’ site from the extracellular side. The ‘Lower’ site is accessible from the intracellular side regardless of whether the SYTANLAAF-gate is open. Our data do not reveal the mechanism underlying relief of Mg^2+^ block by extreme hyperpolarization^9^, which might involve either partial dehydration of the bound Mg^2+^ or permeation of a hydrated Mg^2+^. Also unsolved is the mechanism by which high concentrations of Ca^2+^ attenuate Mg^2+^ block^9^, which could be mediated by competition of Ca^2+^ for Mg^2+^ binding sites, or knock on of bound Mg^2+^ by Ca^2+^ in a multi-ion pore configuration. A recently published Mg^2+^-bound structure in the presence of an antagonist^39^ reported a single Mg^2+^ at a site similar to the ‘Lower’ but not the ‘Upper’ site. This is consistent, as the occupancy of the upper site requires opening of the channel gate, which, in the antagonist-bound structure, is closed^28,43^ and therefore shuts off the entry of extracellular Mg^2+^. Although our intracellular Mg^2+^ binding site is similar in position to the recently reported one, the binding chemistry differs. We observed the intracellular Mg^2+^ in a hydrated form, whereas recent work^39^ proposed partial dehydration by MD simulations. Nonetheless, the distinct existence of the extracellular and intracellular Mg^2+^ sites we observed here aligns with previous electrophysiological findings where the blocking effects of Mg^2+^ from either side were additive^8,35^. The physiological relevance of the intracellular Mg^2+^ block in neuroplasticity remains unclear.

The principle factor for controlling Mg^2+^ binding at the Asn-cage is the membrane electric field and the hydration energy of the Mg^2+^ ion. Our study revealed that two lipids—PL1 and PL2—are bound in the vicinity of the Asn-cage and that the lipid-binding pockets (PL1 and PL2 in Figs. 3–4) are integral parts of the NMDAR protein. A notable feature of our structure is that one acyl chain of PL1 binds deeply around the Asn-cage, while PL2 is located on the back of the Asn-cage and is networked with PL1. Therefore, a subtle change in PL1 and PL2 binding caused by factors such as changes in the electric field may induce local structural changes that could alter the hydrogen bond network of the Asn-cage residues^44^. It is interesting to note that the PL1 binding site is also suggested to be the side-entry vestibule for channel blockers, such as MK801 (refs. ^45,46^), which may imply that the lipid can be interchangeable or movable. The equivalent lipids are absent in other iGluRs, including α-amino-3-hydroxy-5-methyl-4-isoxazolepropionic acid receptors (AMPARs) and kainate receptors. A technically challenging goal would be to capture the Mg^2+^ binding site experimentally at the resting potential, as our MD simulations predicted some changes in Mg^2+^ dynamics in a voltage-dependent manner (Fig. 3c,d). It is also noteworthy that Mg^2+^ block is modulated by the presence of Ca^2+^ and monovalent ions such as Na^+^ (refs. ^7–9^). Elucidating this mechanism will require large datasets and structures at sufficient resolution to capture these ions together with their hydration environments.

**Extended Data Fig. 7:**
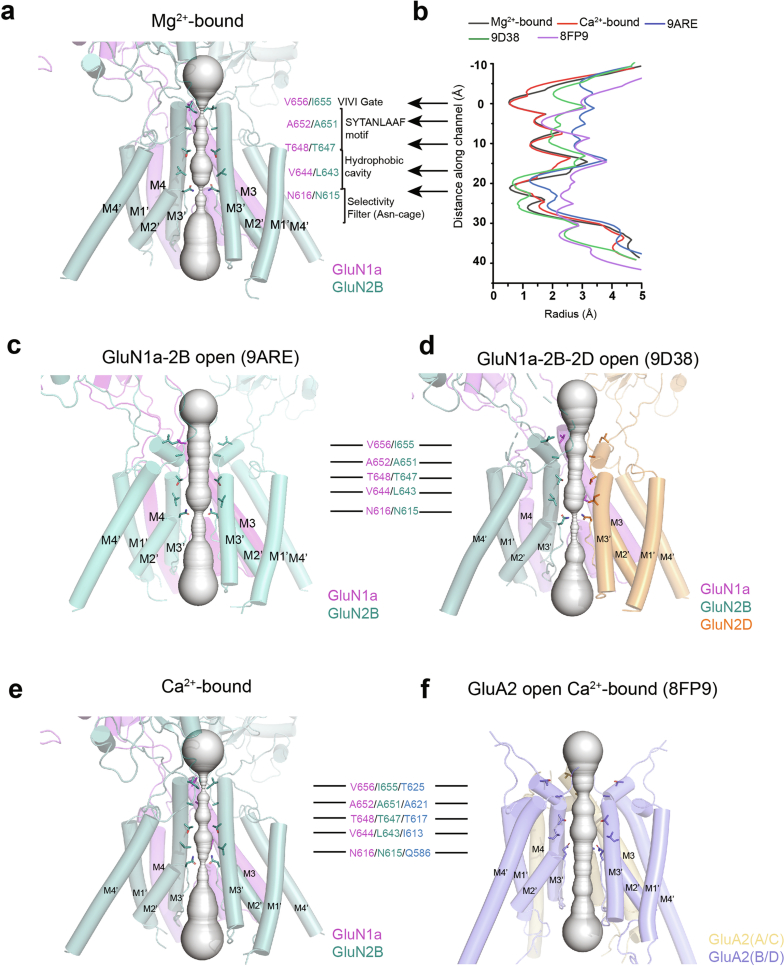
Comparison of iGluR pores. **a-b**, Hole analysis of the GluN1a-2B NMDAR bound to Mg^2+^ and agonists (**a**) and pore-radius measurement (**b**). The TMD motifs are annotated. **c-f**, Hole analysis of GluN1a-2B open channel (PDB code: 9ARE, panel **c**), GluN1a-2B-2D open channel (PDB code: 9D38, panel **d**), Ca^2+^ -bound GluN1a-2B (panel **e**), and GluA2 open channel bound to Ca^2+^ (PDB code: 8FP9, panel **f**).

The architecture of the QRN site in the NMDAR allows Ca^2+^ to become partially dehydrated, conferring Ca^2+^ permeability. It would be meaningful to explore whether the mechanisms underlying Ca^2+^ permeability in NMDAR and the CP-AMPAR are similar. A recent study on the open-state CP-GluA2 AMPAR (Q)/TARP gamma-2 revealed that Ca^2+^ is captured around the channel gate (Site-G) but not at the QNR site^22,47^, potentially indicating the absence of metastable Ca^2+^ binding in its selectivity filter. The existence of Site-G in NMDAR remains unproven. The effort to capture this site in NMDAR in cryo-EM has been hampered by channel-gate closure, possibly due to Ca^2+^ binding to extracellular motifs reported in the past^11,17,48^. The channel pores of CP-GluA2 AMPAR/TARP gamma-2 (ref. ^49^) and GluA1/2 AMPAR/TARP gamma-8 (ref. ^50^) in the open state are substantially larger in diameter than the open-state GluN1a-2B^24^ and GluN1a-2B-2D NMDAR^25^ (Fig. 5b and Extended Data Fig. 7). Indeed, Ca^2+^ permeation through the pore around the QNR site in AMPAR has been demonstrated previously not to involve dehydration by MD simulations^51^. Consistent with this, Mg^2+^, which has a smaller hydration shell dimension than Ca^2+^, has been reported to permeate through CP-GluA2 (ref. ^52^). In contrast, the GluN1a-2B NMDAR QNR site is more constricted than the AMPAR QNR site. Thus, it disallows the passing of Ca^2+^ without dehydration (Fig. 5b). The pore dimension around the QNR site is even smaller in the GluN1a-2B-2D NMDAR (Fig. 5b), consistent with the previous report that the inclusion of GluN2D lowers Ca^2+^ permeability^53^. Therefore, our structural observations suggest that the mechanism of calcium permeability differs between CP-AMPAR and NMDAR^12^. A recent report on the existence of an inner gate around the Asn-cage motif^54^ further complicates the permeability mechanism. Finally, it is essential to note that the Asn-cage residues in GluN2A and GluN2B are susceptible to de novo mutations in humans, which alter Mg^2+^ block and are consequently associated with neurological diseases, including autism spectrum disorder, epilepsy, intellectual disability and developmental delay^55^, indicating the crucial role of the Asn-cage in neuronal functions in brain development.

## Methods

### Expression and purification of GluN1a-2B NMDAR

Rat GluN1a-2B NMDAR was expressed using the EarlyBac system^41^ and purified using a method established previously^56^. Sf9 insect cells at 4.0 × 10^6^ cells ml^−1^ were infected with the recombinant EarlyBac baculovirus harboring both GluN1a and CTD truncated GluN2B (residue 27–852 amino-terminally tagged with a dual strep-tag after the *Xenopus* GluN1 signal peptide and with Cys849Ser). To improve the expression level, 6 of 11 glycosylation sites in GluN1a were mutated as follows: Asn61Gln, Asn239Asp, Asn350Gln, Asn471Gln, Asn491Gln and Asn771Gln. Finally, the endoplasmic reticulum retention signal (Arg/Arg/Lys) at the GluN1a construct was altered by the mutations Arg844Gln, Arg845Gly and Lys846Ala. Cells were collected 48 hours after infection and resuspended in 20 mM HEPES-Na (pH 7.5), 150 mM NaCl, 1 mM glycine, 1 mM Na-glutamate and 1 mM phenylmethylsulfonyl fluoride. Lysis was performed with the aid of Emulsiflex C3 (Avestin). The membrane fraction was obtained by centrifugation at 120,000*g* at 4 °C for 30 min. The membrane-containing pellet was resuspended to a concentration of 100 mg ml^−1^ in buffer containing 20 mM HEPES-Na (pH 7.5), 150 mM NaCl, 1 mM glycine, 1 mM Na-glutamate and 0.5 % LMNG. The desired protein was solubilized for 2 hours at 4 °C. Insoluble material was removed by centrifugation at 120,000*g* for 30 min at 4 °C. Solubilized Strep-tagged GluN1a-2B NMDAR was purified from the supernatant using Strep-tactin Sepharose, followed by SEC (Superose 6 Increase column from GE Healthcare) in buffer containing 20 mM HEPES-Na (pH 7.5), 150 mM NaCl, 0.002% LMNG, 1 mM glycine and 1 mM Na-glutamate. NMDAR containing fractions were concentrated to a protein content of 1–4 mg ml^−1^. The cation-free NMDAR sample was obtained by adding 1 mM EDTA to each buffer during purification. For the cation-bound structures, the ions (10 mM Ca^2+^ and 100 mM Mg^2+^) were added to the purified protein, and the mixture was incubated on ice for 30 min.

### Single-particle cryo-EM analysis on cation-bound GluN1a-2B NMDARs

The cation-free and Ca-bound NMDAR samples were blotted on Quantifoil R 1.2/1.3 + 2 nm C grids (Quantifoil) and Mg-bound NMDAR samples were blotted on UltrAufoil holey gold film grids (Quantifoil) using Leica GP2 at 18 °C and at 85% humidity (blot time, 2.0–3.0 s) and vitrified in liquid ethane. Micrographs were acquired by Titan Krios (FEI) at Cold Spring Harbor Laboratory (CSHL), operating at 300 keV, and the GATAN K3 Summit direct electron detector coupled with the GIF quantum energy filter (Gatan) at ×105,000 magnification (0.827–0.856 Å per pixel), defocus range of −2.4 µm to −0.6 µm, 30 or 40 frames, and 2- to 2.8-second exposure totaling a dose ranging 55.6–71.7 e^−^ Å^−2^. All datasets were processed using Cryosparc v.4.2.1 or v.4.4.1 (ref. ^57^). Collected videos were motion corrected and contrast transfer function-estimated. For particle picking, templates were made from WARP-processed micrographs. Particles were picked using a particle size of 180 Å. Picked particles were extracted and cropped in a 4× box size to enhance the processing speed. Extracted particles were passed through several rounds of two-dimensional classification, ab initio and heterogeneous refinement to reduce the amount of junk particles. Final particle sets were re-extracted to 400–440× box size and refined using nonuniform refinement; 3D classification was used to distinguish between different states. To enhance the overall resolution and local motion correction, the particles were further refined using nonuniform refinement and local refinement (reference-based motion correction). Models were built initially by docking GluN1a-2B (Protein Data Bank (PDB): 7SAA) to the cryo-EM density map using ChimeraX v.1.4 (ref. ^58^). Further processing was done using Phenix v.1.19.2–4158 (ref. ^59^) and refined manually using COOT v.0.9.8.1 (ref. ^60^). A summary of data collection and refinement statistics is shown in Supplementary Table 1.

### Equilibrium MD simulations

Equilibrium MD simulations were performed using OpenMM v.7.5.1 (ref. ^61^). Temperature was set to 298 K using the Langevin Middle integrator with friction coefficient of 1 ps. Pressure was maintained at 1 bar with the Monte Carlo anisotropic barostat. Electrostatics were computed with particle mesh Ewald, with a nonbonded cutoff of 1 nm. Hydrogen masses were repartitioned to 1.5 times their typical molecular weight, allowing for a larger integration step size of 4 fs to be implemented. The protein model consisted of both LBD and TMD and without Cα atom position restraints. For each system, a total of 10 × 200 ns independent simulations were performed.

### Electrophysiology

To express diheteromeric NMDARs in *Xenopus laevis* oocytes for TEVC experiments, we used rGluN1, rGluN2B DNA constructs, as described previously^62^. cRNAs were transcribed using mMessage mMachine t7 transcription kit (Invitrogen) and injected subsequently into defolliculated *X.* *laevis* oocytes (Ecocyte Bioscience) with a total amount of up to 25 ng. Injected oocytes were further incubated in 50% L-15 medium supplemented with 15 mM HEPES pH 7.5, 100× penicillin-streptomycin solution (Thermofisher) and 3 % FBS for 1–2 days at 18 °C. TEVC (Axoclamp-2B) recordings were performed using an extracellular solution containing 5 mM HEPES, 100 mM NaCl, 0.3 mM BaCl_2_ and 10 mM Tricine at a final pH of 7.4 (adjusted with KOH). The current was measured using an agarose-tipped microelectrode (0.4–0.9 MU) at holding potentials ranging from −90 to −20 mV. G/V plots were fitted with the following modified Boltzmann equations:

$$G(V\,)=\frac{G\max }{\left(1+\exp \left[\frac{\left(V-V50\right)z\delta {\rm{F}}}{\mathrm{RT}}\right]\right.}$$

where *V*50 is the half-maximal voltage for Mg^2+^ block, *δ* is the portion of the membrane electric field sensed by the blocking site, *z* is valence, F is Faraday constant, R is gas constant and T is temperature (294.15 K)^34,63^. Maximal response currents were evoked by 100 µM of glycine and 100 µM of L-glutamate. Data were acquired by the PatchMaster program (HEKA) and analyzed using Origin v.8 (OriginLab Corp.). No statistical methods were used to predetermine sample sizes, but our sample sizes are similar to those reported in a previous publication^35^. Data distribution was assumed to be normal, but this was not formally tested. Data collection and analysis were not performed blind to the experimental conditions. No data points were excluded from the analyses.

### Quantification and statistical analysis

Statistical analysis of TEVC and whole-cell patch clamp data was performed using GraphPad Prism and is presented in the corresponding figures and figure legends. Error bars in the fitted curve represent mean ± s.d./s.e.m.; *n* represents the number of independent recordings done on separate cells.

### Reporting summary

Further information on research design is available in the Nature Portfolio Reporting Summary linked to this article.

## Online content

Any methods, additional references, Nature Portfolio reporting summaries, source data, extended data, supplementary information, acknowledgements, peer review information; details of author contributions and competing interests; and statements of data and code availability are available at 10.1038/s41593-026-02283-3.

## Supplementary information

Supplementary Information Supplementary Tables 1–4.

Reporting Summary

Supplementary Video 1 Ca^2+^ permeation pathway in the Asn-cage selectivity filter. This video shows States-1 to -5 captured by single-particle cryo-EM in sequential order.

## Data Availability

Cryo-EM data are deposited with the Electron Microscopy Data Bank (EMDB) and RCSB PDB; Glutamate/glycine-bound GluN1a/2B NMDAR (EMDB: 70297) (PDB: 9OBS), TMD of glutamate/glycine-bound GluN1a/2B NMDAR (EMDB: 70298) (PDB: 9OBT), glutamate/glycine and Ca^2+^-bound GluN1a/2B NMDAR (EMDB: 70302) (PDB: 9OBX), Ca^2+^-bound GluN1a/2B NMDAR (S1) (EMDB: 70303) (PDB: 9OBY), Ca^2+^-bound GluN1a/2B NMDAR (S2) (EMDB: 70304) (PDB: 9OBZ), Ca^2+^-bound GluN1a/2B NMDAR (S3) (EMDB: 70305) (PDB: 9OC0), Ca^2+^-bound GluN1a/2B NMDAR (S4) (EMDB: 70306) (PDB: 9OC1), Ca^2+^-bound GluN1a/2B NMDAR (S5) (EMDB: 70307) (PDB: 9OC2), glycine/glutamate and Mg^2+^-bound GluN1a/2B NMDAR (EMDB: 70299) (PDB: 9OBU), Mg^2+^-bound GluN1a/ 2B NMDAR (lower) (EMDB: 70301) (PDB: 9OBW), Mg^2+^-bound GluN1a/2B NMDAR (upper) (EMDB: 70300) (PDB: 9OBV).

## Code availability

No custom software codes were used in the data analysis for this paper.
