# Supplementary material for: Molecular mechanism of calcium permeability and magnesium block in NMDA receptors
Source: Nat Neurosci. 2026 May 5;29(6):1293–302. doi: 10.1038/s41593-026-02283-3 (PMC13246513; doi:10.1038/s41593-026-02283-3)
Supplement: Supplementary file 1 — Supplementary Tables 1–4. [file 41593_2026_2283_MOESM1_ESM.pdf]

nature neuroscience

Supplementary information

https://doi.org/10.1038/s41593-026-02283-3

Molecular mechanism of calcium permeability and magnesium block in NMDA receptors

In the format provided by the authors and unedited

Supplementary Table 1. Cryo-EM data collection and statistics

	Glutamate/ glycine-bound GluN1a/ 2B NMDAR (EMDB: 70297) (PDB: 9OBS)	TMD of glutamate/glycine-bound GluN1a/2B NMDAR (EMDB: 70298) (PDB: 9OBT)	Glutamate/ glycine and Ca^{2+}-bound GluN1a/2B NMDAR (EMDB: 70302) (PDB: 9OBX)	Ca^{2+}-bound GluN1a/2B NMDAR (S1) (EMDB: 70303) (PDB: 9OBY)	Ca^{2+}-bound GluN1a/2B NMDAR (S2) (EMDB: 70304) (PDB: 9OBZ)	Ca^{2+}-bound GluN1a/2B NMDAR (S3) (EMDB: 70305) (PDB: 9OC0)
**Data collection and processing**						
Microscope	Titan Krios	Titan Krios	Titan Krios	Titan Krios	Titan Krios	Titan Krios
Camera	K3/CDS	K3/CDS	K3/CDS	K3/CDS	K3/CDS	K3/CDS
Magnification	105K	105K	105K	105K	105K	105K
Energy filter slit width (eV)	14	14	14	14	14	14
Collection software	EPU	EPU	EPU	EPU	EPU	EPU
Voltage (kV)	300	300	300	300	300	300
Cumulative exposure (e^-/Å^2)	58.4	58.4	55.6-64.0	58.5	55.6-64.0	55.6-64.0
Exposure rate (e^-/Å^2/frame)	1.46	1.46	1.39-1.6	1.95	1.39-1.6	1.39-1.6
Defocus range (μm)	-2.2~ -0.6	-2.2~ -0.6	-2.2~ -0.6	-2.2~ -0.6	-2.2 ~ -0.6	-2.2 ~ -0.6
Pixel size (Å)	0.827	0.827	0.84	0.827	0.84	0.84
Symmetry imposed	C1	C2	C1	C1	C2	C2
Number of micrographs	12,035	12,035	30,928	18,275	30,928	30,928
Initial particle images (no.)	2,826,035	2,826,035	7,062,525	4,017,866	7,062,525	7,062,525
Final particle images (no.)	131,512	131,512	823,417	55,995	116,195	61,249
0.143 FSC map masked (Å)	3.18	3.01	2.59	3.60	2.81	2.97
0.143 FSC map unmasked(Å)	3.8	4.0	3.1	5.9	3.9	4.2
**Refinement**						
Refinement package	Phenix	Phenix	Phenix	Phenix	Phenix	Phenix
Initial model used (PDB code)	7SAA	7SAA	7SAA	7SAA	7SAA	7SAA
Map sharpening B factor (Å^2)	-99.7	-107.1	-91.2	-105.4	-89.0	-98.0
Model composition						
Non-hydrogen atoms	20,850	3,954	21,017	3,541	4,172	3,987
Protein residues	3154	510	3,160	512	520	512
Ligands	1	6	0	1	7	7
Water	0	28	0	9	39	23
CC map vs. model	0.71	0.64	0.65	0.57	0.63	0.63
R.m.s. deviations						
Bond lengths (Å)	0.005	0.003	0.002	0.007	0.002	0.002
Bond angles (°)	0.516	0.417	0.445	0.999	0.423	0.466
Validation						
MolProbity score	1.83	1.34	1.31	2.16	1.22	1.51
Clashscore	7.10	3.95	2.49	17.26	3.46	4.52
Rotamer outliers (%)	0.84	0.00	0.81	0.65	0.25	0.56
Ramachandran plot						
Favored (%)	93.19	97.12	96.01	93.65	97.58	95.90
Allowed (%)	6.59	2.47	3.90	5.94	2.42	4.10
Outliers (%)	0.22	0.41	0.10	0.41	0.00	0.00
CaBLAM outliers (%)	4.67	1.30	0.00	3.02	1.27	3.88

Supplementary Table 1. Cryo-EM data collection and statistics

	Ca^{2+}-bound GluN1a/2B NMDAR (S4) (EMDB: 70306) (PDB: 9OC1)	Ca^{2+}-bound GluN1a/2B NMDAR (S5) (EMDB: 70307) (PDB: 9OC2)	Glycine/glutamate and Mg^{2+}-bound GluN1a/2B NMDAR (EMDB: 70299) (PDB: 9OBU)	Mg^{2+}-bound GluN1a/2B NMDAR (lower) (EMDB: 70301) (PDB: 9OBW)	Mg^{2+}-bound GluN1a/2B NMDAR (upper) (EMDB: 70300) (PDB: 9OBV)
**Data collection and processing**					
Microscope	Titan Krios	Titan Krios	Titan Krios	Titan Krios	Titan Krios
Camera	K3/CDS	K3/CDS	K3/CDS	K3/CDS	K3/CDS
Magnification	105K	105K	105K	105K	105K
Energy filter slit width (eV)	14	14	14-20	14-20	14-20
Collection software	EPU	EPU	EPU	EPU	EPU
Voltage (kV)	300	300	300	300	300
Cumulative exposure ($e^-/Å^2$)	55.6-64.0	55.6-64.0	58.2-71.7	58.2-71.7	58.2-71.7
Exposure rate ($e^-/Å^2$/frame)	1.39-1.6	1.39-1.6	1.94-2.39	1.94-2.39	1.94-2.39
Defocus range (μm)	-2.2~ -0.6	-2.2~ -0.6	-2.6~ -0.8	-2.6~ -0.8	-2.6 ~ -0.8
Pixel size (Å)	0.84	0.84	0.856	0.856	0.856
Symmetry imposed	C2	C2	C1	C2	C2
Number of micrographs	30,928	30,928	38,717	38,717	38,717
Initial particle images (no.)	7,062,525	7,062,525	5,106,550	5,106,550	5,106,550
Final particle images (no.)	102,122	87,601	512,800	118,618	105,462
0.143 FSC map masked (Å)	2.76	2.69	3.16	3.15	3.21
0.143 FSC map unmasked(Å)	4.0	4.0	3.6	4.1	4.1
**Refinement**					
Refinement package	Phenix	Phenix	Phenix	Phenix	Phenix
Initial model used (PDB code)	7SAA	7SAA	7SAA	7SAA	7SAA
Map sharpening B factor ($Å^2$)	-78.6	-88.0	-118.8	-127.0	-115.4
Model composition					
Non-hydrogen atoms	4,031	3950	21,219	3,762	3,850
Protein residues	512	510	3161	500	508
Ligands	7	7	0	5	5
Water	36	25	0	26	27
CC map vs. model	0.62	0.63	0.68	0.66	0.60
R.m.s. deviations					
Bond lengths (Å)	0.002	0.003	0.004	0.004	0.003
Bond angles (°)	0.471	0.435	0.496	0.465	0.429
Validation					
MolProbity score	1.53	1.25	1.49	1.13	1.37
Clashscore	5.96	4.81	3.54	2.31	5.14
Rotamer outliers (%)	0.00	0.00	0.36	0.62	0.60
Ramachandran plot					
Favored (%)	96.72	98.77	94.96	97.48	97.52
Allowed (%)	3.28	2.44	1.23	2.31	2.48
Outliers (%)	0.00	0.00	0.00	0.21	0.00
CaBLAM outliers (%)	1.72	1.92	0.87	0.66	2.17

Supplementary Table 2. IC_{50}, V_{50}, and δ values of GluN1-4a N616Q and GluN2B N615Q mutants

	IC_{50} [μM] at -60 mV			
WT	16.8 ± 2.1	n_H = 1.02 ± 0.15	n = 5	
GluN1-4a N616Q	1614 ± 218	n_H = 0.45 ± 0.02	n = 4	p = <0.0001
GluN2B N615Q	1148 ± 111	n_H = 0.80 ± 0.02	n = 4	p = <0.0001

IC_{50} values ± SD derived from Mg^{2+} concentration-response curves at -60, -40, -20 mV through TEVC. Statistical significance was assessed using one-way analysis of variance (ANOVA) followed by Dunnett's test (two-sided) comparing each group with the WT. P values were adjusted for multiple comparisons with family-wise α = 0.05. The table lists the IC_{50} values, and Hill coefficients ± SD (n_H) calculated based on the dose-response curves. IC_{50} values were calculated from independent dose-response recordings from at least four independent oocytes (n). The isotype of GluN1a used in these TEVC experiments is GluN1-4a.

[Mg^{2+}]	0.1 mM	0.25 mM	0.5 mM
WT	V_{50} = -38.0 ± 1.4<br>δ = 1.24 ± 0.05<br>n = 6	V_{50} = -30.7 ± 1.5<br>δ = 1.43 ± 0.04<br>n = 6	V_{50} = -26.2 ± 1.8<br>δ = 1.65 ± 0.04<br>n = 6
GluN1-4a N616Q	V_{50} = -53.1 ± 0.4<br>p_{V50} = <0.0001<br>δ = 0.74 ± 0.03<br>$p_δ$ = <0.0001<br>n = 10	V_{50} = -43.9 ± 0.6<br>p_{V50} = <0.0001<br>δ = 0.89 ± 0.03<br>$p_δ$ = <0.0001<br>n = 10	V_{50} = -34.2 ± 0.8<br>p_{V50} = 0.0032<br>δ = 1.01 ± 0.04<br>$p_δ$ = <0.0001<br>n = 10
GluN2B N615Q	V_{50} = -74.8 ± 2.2<br>p_{V50} = <0.0001<br>δ = 0.93 ± 0.07<br>$p_δ$ = 0.0004<br>n = 8	V_{50} = -60.3 ± 1.7<br>p_{V50} = <0.0001<br>δ = 0.96 ± 0.04<br>$p_δ$ = <0.0001<br>n = 8	V_{50} = -48.4 ± 1.7<br>p_{V50} = <0.0001<br>δ = 1.00 ± 0.01<br>$p_δ$ = <0.0001<br>n = 8

V_{50} and δ values ± SE derived from varying Mg^{2+} concentration I/V curves (0.1, 0.25, 0.5 mM) through TEVC. Statistical significance was assessed using one-way analysis of variance (ANOVA) followed by Dunnett's test (two-sided) comparing each group with the WT. P values were adjusted for multiple comparisons with family-wise α = 0.05. The table lists the V_{50} values, and δ ± SE calculated based on the I/V curves. V_{50} values were calculated from independent I/V recordings from at least three independent oocytes (n). The isotype of GluN1a used in these TEVC experiments is GluN1-4a.

Supplementary Table 3. IC_{50} [μM] values of lipid-binding site mutants

[mV]	-60	-40	-20
WT	16.8 ± 2.1<br>n_H = 0.94 ± 0.03 ; n = 5	79.1 ± 4.5<br>n_H = 0.88 ± 0.02 ; n = 5	1662 ± 52<br>n_H = 0.78 ± 0.06 ; n = 6
GluN1-4a V566W	44.9 ± 4.4<br>p = <0.0001<br>n_H = 0.81 ± 0.10 ; n = 6	243.9 ± 11.2<br>p = <0.0001<br>n_H = 0.75 ± 0.05 ; n = 5	1042 ± 236<br>p = 0.1422<br>n_H = 1.13 ± 0.23 ; n = 4
GluN1-4a G567W	35.0 ± 4.3<br>p = <0.0001<br>n_H = 0.84 ± 0.06 ; n = 5	201.8 ± 18.8<br>p = <0.0001<br>n_H = 0.84 ± 0.13 ; n = 4	4989 ± 488<br>p = <0.0001<br>n_H = 1.62 ± 0.10 ; n = 5
GluN1-4a S604W	20.9 ± 1.6<br>p = 0.2759<br>n_H = 0.93 ± 0.02 ; n = 5	91.9 ± 4.0<br>p = 0.5708<br>n_H = 0.88 ± 0.02 ; n = 5	5915 ± 342<br>p = <0.0001<br>n_H = 1.14 ± 0.03 ; n = 4
GluN1-4a M607W	41.6 ± 1.2<br>p = <0.0001<br>n_H = 0.93 ± 0.04 ; n = 5	174.2 ± 12.6<br>p = <0.0001<br>n_H = 0.80 ± 0.02 ; n = 5	7041 ± 677<br>p = <0.0001<br>n_H = 1.77 ± 0.12 ; n = 5
GluN1-4a L615W	24.4 ± 1.9<br>p = 0.0147<br>n_H = 0.99 ± 0.05 ; n = 4	105.3 ± 3.5<br>p = 0.0601<br>n_H = 0.94 ± 0.04 ; n = 4	1018 ± 146<br>p = 0.0671<br>n_H = 0.95 ± 0.11 ; n = 6
GluN1-4a L615Q	48.7 ± 3.8<br>p = <0.0001<br>n_H = 0.97 ± 0.03 ; n = 6	264.6 ± 23.8<br>p = <0.0001<br>n_H = 0.91 ± 0.06 ; n = 4	2365 ± 370<br>p = 0.0763<br>n_H = 1.48 ± 0.13 ; n = 4
GluN2B T626W	14 ± 1.9<br>p = 0.9191<br>n_H = 0.93 ± 0.06 ; n = 4	89.7 ± 5.0<br>p = 0.999<br>n_H = 0.85 ± 0.02 ; n = 5	2176 ± 490<br>p = 0.1448<br>n_H = 1.04 ± 0.04 ; n = 5
GluN2B I630W	23.4 ± 1.7<br>p = 0.3216<br>n_H = 1.02 ± 0.07 ; n = 5	79.3 ± 2.9<br>p = >0.9999<br>n_H = 0.92 ± 0.02 ; n = 4	5655 ± 476<br>p = <0.0001<br>n_H = 1.68 ± 0.06 ; n = 4
GluN2B S633L	164.3 ± 11.4<br>p = <0.0001<br>n_H = 0.80 ± 0.04 ; n = 5	3015 ± 184<br>p = <0.0001<br>n_H = 0.82 ± 0.03 ; n = 4	10472 ± 1068<br>p = <0.0001<br>n_H = n.d. ; n = 5
GluN2B F637W	26.9 ± 2.3<br>p = 0.0905<br>n_H = 1.03 ± 0.04 ; n = 4	95.6 ± 10.2<br>p = 0.9946<br>n_H = 1.10 ± 0.06 ; n = 5	661.7 ± 29.4<br>p = 0.0614<br>n_H = 1.57 ± 0.06 ; n = 4

IC_{50} values ± SD derived from Mg^{2+} concentration-response curves at -60, -40, -20 mV through TEVC. Statistical significance was assessed using one-way analysis of variance (ANOVA) followed by Dunnett's test (two-sided) comparing each group with the WT. P values were adjusted for multiple comparisons with family-wise α = 0.05. The table lists the IC_{50} values, and Hill coefficients ± SD (n_H) calculated based on the dose-response curves. IC_{50} values were calculated from independent dose-response recordings from at least four independent oocytes (n). The isotype of GluN1a used in these TEVC experiments is GluN1-4a.

Supplementary Table 4. V_{50} and δ values of lipid-binding site mutants

$[Mg^{2+}]$	0.1 mM	0.25 mM	0.5 mM
WT	V_{50} = -38.0 ± 1.4<br>δ = 1.24 ± 0.05<br>n = 6	V_{50} = -30.7 ± 1.5<br>δ = 1.43 ± 0.04<br>n = 6	V_{50} = -26.2 ± 1.8<br>δ = 1.65 ± 0.04<br>n = 6
GluN1-4a V566W	V_{50} = -48.0 ± 2.3<br>p_{V50} = <0.0001<br>δ = 1.49 ± 0.12<br>$p_δ$ = 0.0185<br>n = 5	V_{50} = -40.2 ± 2.7<br>p_{V50} = 0.0008<br>δ = 1.64 ± 0.06<br>$p_δ$ = 0.0086<br>n = 5	V_{50} = -35.5 ± 3.5<br>p_{V50} = 0.0035<br>δ = 1.67 ± 0.05<br>$p_δ$ = 0.9996<br>n = 5
GluN1-4a G567W	V_{50} = -48.7 ± 0.9<br>p_{V50} = <0.0001<br>δ = 1.02 ± 0.03<br>$p_δ$ = 0.0355<br>n = 7	V_{50} = -38.8 ± 1.5<br>p_{V50} = 0.0032<br>δ = 1.12 ± 0.03<br>$p_δ$ = <0.0001<br>n = 7	V_{50} = -31.5 ± 1.3<br>p_{V50} = 0.1821<br>δ = 1.23 ± 0.03<br>$p_δ$ = <0.0001<br>n = 7
GluN1-4a S604W	V_{50} = -47.3 ± 0.4<br>p_{V50} = <0.0001<br>δ = 1.11 ± 0.03<br>$p_δ$ = 0.4112<br>n = 6	V_{50} = -37.2 ± 0.5<br>p_{V50} = 0.0270<br>δ = 1.20 ± 0.03<br>$p_δ$ = 0.0020<br>n = 6	V_{50} = -31.2 ± 0.4<br>p_{V50} = 0.2321<br>δ = 1.33 ± 0.02<br>$p_δ$ = <0.0001<br>n = 6
GluN1-4a M607W	V_{50} = -50.7 ± 0.4<br>p_{V50} = <0.0001<br>δ = 1.23 ± 0.01<br>$p_δ$ = 0.9997<br>n = 6	V_{50} = -41.6 ± 0.5<br>p_{V50} = <0.0001<br>δ = 1.31 ± 0.01<br>$p_δ$ = 0.2785<br>n = 6	V_{50} = -35.5 ± 0.4<br>p_{V50} = 0.0021<br>δ = 1.39 ± 0.02<br>$p_δ$ = 0.0008<br>n = 6
GluN1-4a L615W	V_{50} = -31.0 ± 0.8<br>p_{V50} = 0.0039<br>δ = 1.30 ± 0.02<br>$p_δ$ = 0.9716<br>n = 6	V_{50} = -25.8 ± 0.4<br>p_{V50} = 0.1464<br>δ = 1.59 ± 0.01<br>$p_δ$ = 0.0534<br>n = 6	V_{50} = -22.5 ± 0.5<br>p_{V50} = 0.5626<br>δ = 1.82 ± 0.02<br>$p_δ$ = 0.0442<br>n = 6
GluN1-4a L615Q	V_{50} = -53.9 ± 2.8<br>p_{V50} = <0.0001<br>δ = 1.22 ± 0.04<br>$p_δ$ = 0.9997<br>n = 3	V_{50} = -45.6 ± 2.7<br>p_{V50} = <0.0001<br>δ = 1.34 ± 0.06<br>$p_δ$ = 0.8262<br>n = 3	V_{50} = -39.2 ± 5.2<br>p_{V50} = 0.0004<br>δ = 1.36 ± 0.21<br>$p_δ$ = 0.0019<br>n = 3
GluN1-4a N616Q	V_{50} = -53.1 ± 0.6<br>p_{V50} = <0.0001<br>δ = 0.61 ± 0.02<br>$p_δ$ = <0.0001<br>n = 10	V_{50} = -43.9 ± 1.5<br>p_{V50} = <0.0001<br>δ = 0.64 ± 0.05<br>$p_δ$ = <0.0001<br>n = 10	V_{50} = -34.2 ± 1.5<br>p_{V50} = 0.0032<br>δ = 0.83 ± 0.01<br>$p_δ$ = <0.0001<br>n = 10
GluN2B I630W	V_{50} = -39.1 ± 1.3<br>p_{V50} = 0.9969<br>δ = 1.12 ± 0.05<br>$p_δ$ = 0.4910<br>n = 7	V_{50} = -31.1 ± 0.9<br>p_{V50} = 0.9998<br>δ = 1.35 ± 0.04<br>$p_δ$ = 0.7382<br>n = 7	V_{50} = -26.9 ± 0.8<br>p_{V50} = 0.9996<br>δ = 1.52 ± 0.04<br>$p_δ$ = 0.2381<br>n = 7
GluN2B S633L	V_{50} = -42.8 ± 0.9<br>p_{V50} = 0.1734<br>δ = 1.15 ± 0.09<br>$p_δ$ = 0.8677<br>n = 4	V_{50} = -33.4 ± 1.4<br>p_{V50} = 0.8239<br>δ = 1.53 ± 0.04<br>$p_δ$ = 0.4714<br>n = 4	V_{50} = -27.6 ± 0.8<br>p_{V50} = 0.9967<br>δ = 1.61 ± 0.07<br>$p_δ$ = 0.9924<br>n = 4

GluN2B N615Q	V_{50} = -74.8 ± 2.2<br>p_{V50} = <0.0001<br>δ = 0.93 ± 0.07<br>p_{δ} = 0.0004<br>n = 8	V_{50} = -60.3 ± 1.7<br>p_{V50} = <0.0001<br>δ = 0.96 ± 0.04<br>p_{δ} = <0.0001<br>n = 8	V_{50} = -48.4 ± 1.7<br>p_{V50} = <0.0001<br>δ = 1.00 ± 0.01<br>p_{δ} = <0.0001<br>n = 8

V_{50} and δ values ± SE derived from varying Mg^{2+} concentration I/V curves (0.1, 0.25, 0.5 mM) through TEVC. Statistical significance was assessed using one-way analysis of variance (ANOVA) followed by Dunnett's test (two-sided) comparing each group with the WT. P values were adjusted for multiple comparisons with family-wise α = 0.05. The table lists the V_{50} values, and δ ± SE calculated based on the I/V curves. V_{50} values were calculated from independent I/V recordings from at least three independent oocytes (n). The isotype of GluN1a used in these TEVC experiments is GluN1-4a.
