# Supplementary material for: Molecular mechanism of calcium permeability and magnesium block in NMDA receptors
Source: Nat Neurosci. 2026 May 5;29(6):1293–302. doi: 10.1038/s41593-026-02283-3 (PMC13246513; doi:10.1038/s41593-026-02283-3)
Supplement: Supplementary file 2 — Reporting Summary [file 41593_2026_2283_MOESM2_ESM.pdf]

nature portfolio

Corresponding author(s): Hiro Furukawa

Last updated by author(s): Mar 12, 2026

Reporting Summary

Nature Portfolio wishes to improve the reproducibility of the work that we publish. This form provides structure for consistency and transparency in reporting. For further information on Nature Portfolio policies, see our Editorial Policies and the Editorial Policy Checklist.

Statistics

For all statistical analyses, confirm that the following items are present in the figure legend, table legend, main text, or Methods section.

n/a	Confirmed	
☐	☒	The exact sample size (*n*) for each experimental group/condition, given as a discrete number and unit of measurement
☐	☒	A statement on whether measurements were taken from distinct samples or whether the same sample was measured repeatedly
☐	☒	The statistical test(s) used AND whether they are one- or two-sided<br>*Only common tests should be described solely by name; describe more complex techniques in the Methods section.*
☒	☐	A description of all covariates tested
☐	☒	A description of any assumptions or corrections, such as tests of normality and adjustment for multiple comparisons
☐	☒	A full description of the statistical parameters including central tendency (e.g. means) or other basic estimates (e.g. regression coefficient) AND variation (e.g. standard deviation) or associated estimates of uncertainty (e.g. confidence intervals)
☐	☒	For null hypothesis testing, the test statistic (e.g. *F*, *t*, *r*) with confidence intervals, effect sizes, degrees of freedom and *P* value noted<br>*Give P values as exact values whenever suitable.*
☒	☐	For Bayesian analysis, information on the choice of priors and Markov chain Monte Carlo settings
☒	☐	For hierarchical and complex designs, identification of the appropriate level for tests and full reporting of outcomes
☒	☐	Estimates of effect sizes (e.g. Cohen's *d*, Pearson's *r*), indicating how they were calculated

Our web collection on statistics for biologists contains articles on many of the points above.

Software and code

Policy information about availability of computer code

Data collection	EPU 2.10.0.5 was used for Cryo-EM data collection. PatchMaster v2x32 softwares were used for electrophysiological data collection.
Data analysis	CryoSPARC 3.2.0 were used for Cryo-EM single-particle analysis. PHENIX 1.15.2, Coot 0.8.9, Chimera 1.11.2 and Pymol 2.3.1 were used for model building, refinement and analysis. OriginPro 2022b were used for electrophysiological data analysis. For MD simulation, Modeller 9.12, Antechamber, Gromacs 2021.3, Python 3.0, and OpenMM version 7.5.1 were used for fixing residues, ligand parameterization, setting up/ performing simulations, analysis, and unbiased simulations, respectively.

For manuscripts utilizing custom algorithms or software that are central to the research but not yet described in published literature, software must be made available to editors and reviewers. We strongly encourage code deposition in a community repository (e.g. GitHub). See the Nature Portfolio guidelines for submitting code & software for further information.

Data

Policy information about availability of data

All manuscripts must include a data availability statement. This statement should provide the following information, where applicable:

- Accession codes, unique identifiers, or web links for publicly available datasets
- A description of any restrictions on data availability
- For clinical datasets or third party data, please ensure that the statement adheres to our policy

Cryo-EM density maps have been deposited in the Electron Microscopy Data Bank (EMDB) under accession codes ### and @@@. The structural coordinates have been deposited in the RCSB Protein Data Bank (PDB) under accession codes $$ and ++.

Research involving human participants, their data, or biological material

Policy information about studies with human participants or human data. See also policy information about sex, gender (identity/presentation), and sexual orientation and race, ethnicity and racism.

Reporting on sex and gender	n.a.
Reporting on race, ethnicity, or other socially relevant groupings	n.a.
Population characteristics	n.a.
Recruitment	n.a.
Ethics oversight	n.a.

Note that full information on the approval of the study protocol must also be provided in the manuscript.

Field-specific reporting

Please select the one below that is the best fit for your research. If you are not sure, read the appropriate sections before making your selection.

☒ Life sciences ☐ Behavioural & social sciences ☐ Ecological, evolutionary & environmental sciences

For a reference copy of the document with all sections, see nature.com/documents/nr-reporting-summary-flat.pdf

Life sciences study design

All studies must disclose on these points even when the disclosure is negative.

Sample size	Sample sizes were no predetermined in this study. The sample sizes of Cryo-EM data were governed by the availability of the microscope. The data of the electrophysiological experiments were repeated at least six times.
Data exclusions	In cryo-EM single-particle analysis, particles in 2D and 3D classes which did not possess high-resolution features were removed in the final 3D reconstruction. The additional 3D classification focused on the TMD was done on channel blocker bound samples to exclude the non-ligand bound particles.
Replication	Cryo-EM related experiments including protein expression, purification were reproduced at least three times independently. Cryo-EM data collections for each condition were performed at least two times in two different dates. Electrophysiology experiments were repeated at least on six different oocytes.
Randomization	In cryo-EM related experiments, all micrograph movies were acquired in random places on the EM grids and particles were randomly partitioned for resolution and quality assessment in single-particle analysis. Electrophysiological experiments were not randomized.
Blinding	The investigators were not blinded. Blinding serves no purpose for cryo-EM single-particle analysis, MD simulation and electrophysiological experiments.

Reporting for specific materials, systems and methods

We require information from authors about some types of materials, experimental systems and methods used in many studies. Here, indicate whether each material, system or method listed is relevant to your study. If you are not sure if a list item applies to your research, read the appropriate section before selecting a response.

Materials & experimental systems

n/a	Involved in the study	
☒	☐	Antibodies
☐	☒	Eukaryotic cell lines
☒	☐	Palaeontology and archaeology
☒	☐	Animals and other organisms
☒	☐	Clinical data
☒	☐	Dual use research of concern
☒	☐	Plants

Methods

n/a	Involved in the study	
☒	☐	ChIP-seq
☒	☐	Flow cytometry
☒	☐	MRI-based neuroimaging

Eukaryotic cell lines

Policy information about cell lines and Sex and Gender in Research

Cell line source(s)	Sf9 insect cells used in the study were provided by Cold Spring Harbor Laboratory. Xenopus oocytes were either harvested from the frogs raised in the animal facilities of Cold Spring Harbor Laboratory or purchased from Ecocyte Bioscience.
Authentication	The cells were routinely maintained in our laboratory They were not authenticated for these cell lines.
Mycoplasma contamination	The cell lines were tested negative for mycoplasma contamination.
Commonly misidentified lines (See ICLAC register)	No commonly misidentified lines were used in this study.

Plants

Seed stocks	n.a.
Novel plant genotypes	n.a.
Authentication	n.a.
